# Neuroprotective effect of Astragali Radix on cerebral infarction based on proteomics

**DOI:** 10.3389/fphar.2023.1162134

**Published:** 2023-06-09

**Authors:** Ying Li, Daoping Wang, Rongjuan Guo, Bo Ma, Lan Miao, Mingqian Sun, Lijuan He, Li Lin, Yinghong Pan, Junguo Ren, Jianxun Liu

**Affiliations:** ^1^ Beijing Key Laboratory of Pharmacology of Chinese Materia Region, Xiyuan Hospital, Institute of Basic Medical Sciences, National Clinical Research Center of Cardiovascular Disease of Traditional Chinese Medicine, China Academy of Chinese Medical Sciences, Beijing, China; ^2^ Institute of Crop Sciences, Chinese Academy of Agricultural Sciences, Beijing, China; ^3^ Dongfang Hospital, Beijing University of Chinese Medicine, Beijing, China; ^4^ Institute of Materia Medica, Chinese Academy of Medical Science and Peking Union Medical College, Beijing, China

**Keywords:** cerebral infarction, proteomics, neuroprotective, mechanism, Astragali Radix

## Abstract

**Objective:** Astragali Radix (AR, Huangqi in Chinese) has a neuroprotective effect on cerebral infarction (CI). In order to explore the biological basis and therapeutic mechanism of AR in CI, a double-blind randomized controlled trial was established in this study, and proteomics analysis was carried out on serum samples of patients.

**Methods:** The patients were divided into the AR group (*n* = 35) and the control group (*n* = 30). The curative effect was evaluated by the traditional Chinese medicine (TCM) syndrome score and clinical indicators, and the serum of the two groups was analyzed by proteomics. Based on bioinformatics analysis methods, the changes in differential proteins between two groups of samples were explored, and the key proteins were validated through enzyme-linked immunosorbent assay (ELISA).

**Results:** The results of this study showed that the scores of deficiency of vital energy (DVE), blood stasis (BS), and NIH Stroke Scale (NIHSS) decreased significantly (*p* < 0.05), while the scores of the Barthel Index (BI) increased, indicating that AR could significantly improve the symptoms of CI patients. In addition, we found that compared with the control group, AR upregulated 43 proteins and downregulated 20 proteins, especially focusing on anti-atherosclerosis and neuroprotective effects. Moreover, ELISA indicated the levels of IL-6, TNF-α, VCAM-1, MCP-1, and ICAM-1 were significantly decreased in the serum of the AR group (*p* < 0.05, *p* < 0.01).

**Conclusion:** This study found that AR can significantly recover the clinical symptoms of CI. Serum proteomics research results show that AR may act on IL-6, TNF-α, VCAM-1, MCP-1, and ICAM-1, and play anti-atherosclerosis and neuroprotective roles.

**Clinical Trial Registration:** [clinicaltrials.gov], identifier [NCT02846207]

## Introduction

Clinically, cerebral infarction (CI) or ischemic stroke is linked to cell death induced by ischemia and hypoxia of local brain tissue caused by vasospasm, stenosis, or occlusion due to local lesions of the vascular wall (Fan et al., 2020; Hui et al., 2021; Wang et al., 2020). It is reported that CI is one of the most common causes of death in the world. According to the most recent prevalence statistics from the American Heart Association, it is estimated that 5,400,000 people have experienced a stroke (Zhou et al., 2019). With the global aging population, an estimated 23 million people suffer from stroke and 7.8 million people will die from 2002 to 2030. About 3/4 of the survivors are incapacitated to varying degrees, and the severe disability rate will be 40% or higher (Kleindorfer et al., 2021; Mathers and Loncar, 2006). Cohort studies have shown that with the improvement of stroke treatment strategies, mortality has dropped. However, the prevalence of recurrent stroke still deserves attention, and effective means are needed to prevent and alleviate secondary stroke (Boot et al., 2020; Ma et al., 2021; Shen et al., 2022). Therefore, it is vital to effectively improve the quality of life and long-term prognosis of CI, and to prevent and alleviate secondary stroke.

In cerebral ischemia, cerebrovascular obstruction deprives brain cells of essential nutrients and oxygen, leading to severe neurological deficits. Ischemic injury may lead to irreversible injury or death of ischemic core neurons. Ischemic stroke involves many mechanisms, such as neurotoxicity, mitochondrial dysfunction, oxidative stress, inflammation, and apoptosis (Chen et al., 2020). These pathophysiological mechanisms overlap and correlate with the development of ischemic stroke and are potential pharmacological targets for treating ischemic stroke.

Chinese herbal medicine has been applied in the treatment of cerebral ischemia for thousands of years and has accumulated much clinical experience (Li et al., 2016; Seto et al., 2016). Astragali Radix (AR, Huangqi in Chinese), the dried root of *Astragalus membranaceus* (Fisch.) Bge. var. mongholicus (Bge.) Hsiao or *A. membranaceus* (Fisch.) Bge., which was first recorded in the Shennong Materia Medica (220–280 AD) and was used for ischemic stroke since Tang Dynasty (Gong et al., 2018). Pharmacological studies have found that AR contains a variety of bioactive substances, including saponins, polysaccharides, flavonoids, amino acids, and trace elements (Fu et al., 2014).

Proteomics technology has been widely used in discovering potential protein molecular markers and drug targets of diseases, and exploring protein action mechanisms of action (Tewari et al., 2018). Numerous studies have shown that proteomics technology can be used to explore the proteins related to diseases and reveal their pathogenesis (Chen et al., 2021; Yang et al., 2021; Zhang et al., 2021). So what is AR’s mechanism in treating stroke and what essential proteins will be regulated to play a therapeutic role?

**FIGURE 1:**
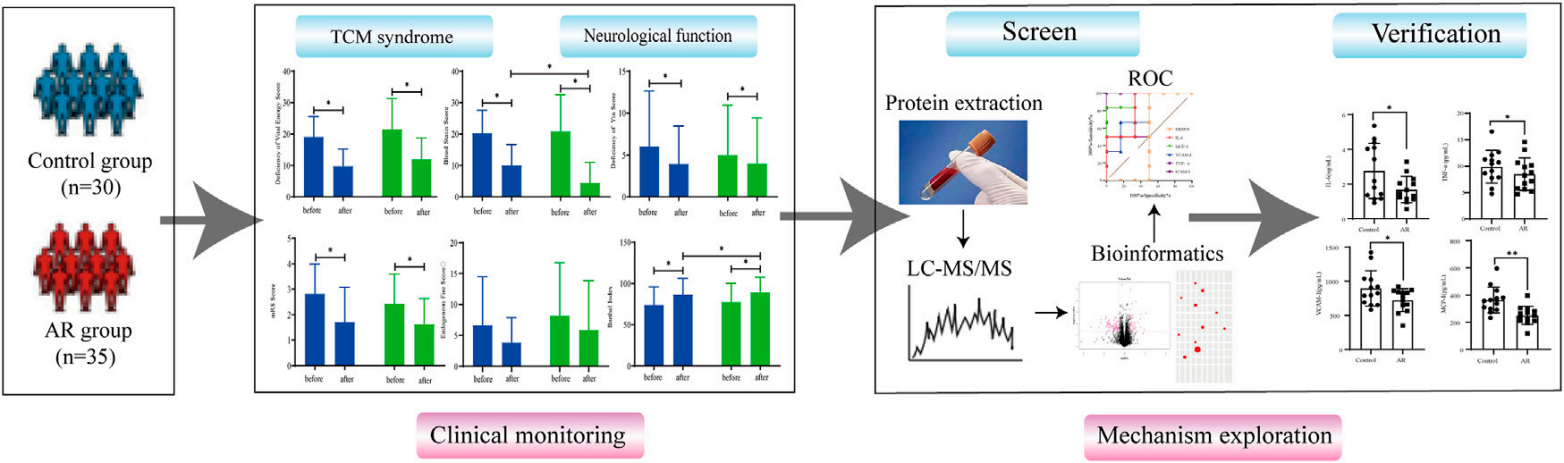
Flowchart of the study.

In this study, a randomized, double-blind, placebo-controlled clinical trial was conducted to evaluate the effectiveness of AR in treating CI. Then, the difference in the serum protein content was analyzed using proteomics technology to explore further the neuroprotective effect and potential target protein of AR on ischemic stroke (See Figure 1 for the flowchart of this study).

## Materials and methods

### Patient population

Overall, a randomized, double-blind, placebo-controlled, parallel-group clinical study was conducted in China from January 2016 to December 2018. This study recruited 65 patients who met the inclusion and exclusion criteria, according to the 2013 Guidelines for the Early Management of Patients with Acute Ischemic Stroke from the American Heart Association/American Stroke Association and the Chinese guidelines for diagnosis and treatment of acute ischemic stroke 2014. In brief, diagnostic inclusion criteria include 1) age 40 to 85; 2) Qi deficiency and blood stasis (BS) syndrome (Ren et al., 2012); 3) accord with the diagnostic criteria of atherosclerotic thrombotic CI; carotid artery color Doppler ultrasound showed evidence of atherosclerotic plaque, and CT/MRI showed definite infarct; 4) NIHSS scores range from 3 to 22; 5) the course of the disease was in the recovery period (2 weeks–6 months); and 6) submitted informed consent. (See Supplementary Material S1 for specific inclusion and exclusion criteria).

### Interventions

The composition of AR granules was 60 g of *Astragalus membranaceus* (Fisch.) Bge. var. mongholicus (Bge.) Hsiao, which was provided by China Resources Sanjiu Pharmaceutical Co., Ltd., and the batch number was 1812002C. By using the method of liquid chromatography tandem mass spectrometry (LC-MS/MS), we identified eight metabolites from the AR (Please see Supplementary Material S2 for more information). Patients with CI were randomly divided into the Astragali Radix group (AR) and the control group (Con). Both groups received primary treatment in the convalescent stage of CI (refer to Chinese guidelines for diagnosing and treating acute ischemic stroke 2014). Based on the standard treatment of Western medicine, the AR and placebo were added. The placebo group was given a traditional Chinese medicine (TCM) simulator. The patient takes AR orally twice a day, with one pack administered each time. The entire course of the treatment lasts for 12 weeks. All patients were suspended from using TCM related to the treatment of stroke treatment 1 week in advance. The randomization procedure was designed by the Data Manager at Xiyuan Hospital of China Academy of Chinese Medical Sciences using SAS statistical software.

### Outcome assessments

The primary efficacy endpoint included TCM syndrome and neurological function scores, including the NIH Stroke Scale (NIHSS) and Barthel Index (BI). The efficacy criteria were developed according to the principles of clinical research. The secondary efficacy endpoint includes total cholesterol (TC), triglyceride (TG), high-density lipoprotein cholesterol (HDL-C), and low-density lipoprotein cholesterol (LDL-C). The safety endpoints include blood, liver, and kidney function tests, and record the aforementioned endpoints once. The adverse event (AE)/adverse drug reaction (ADR) record form was truthfully completed during the trial.

### Preparation of serum samples

The subjects’ whole blood was collected and centrifuged at 1,500 *g* for 15 min to obtain the serum. The upper serum was carefully sucked out and stored at −80°C. In each group, a total of six biological replicates for each of the groups were used for proteomics analysis, including three men and three women. The serum was thawed, and 50 μl was taken as a sample for subsequent processing. Albumin and IgG of the serum were depleted using a ProteoExtract Albumin/IgG Removal Kit (Merck, Cat. No. 122642). The serum was transferred to a 10-kDa ultrafiltration tube, and dithiothreitol (DTT, Thermo Fisher, Cat. No.3483-12-3) was added to a final concentration of 50 mmol/L and placed at 37 °C for 30 min. Subsequently, 50 mmol/L urea (Affymetrix, 57–13–6) was replaced thrice. The final concentration of iodoacetamide (IAA, GE, Cat. No.I1149) was 100 mmol/L and placed in the dark at 37°C for 30 min, and then replaced with 50 mmol/L urea and ammonium bicarbonate (NH_4_CO_3_, Amresco, Cat. No.1066-33-7) solution three times. The proteins were then digested with trypsin (Promega, Cat. No. V5111, enzyme/protein ratio of 1:25 w/w) at 37°C for rocking overnight. Furthermore, 0.1% formic acid (FA, Thermo Fisher, Cat. No.214241) was added to terminate the reaction, and the receiver tube was replaced and centrifuged at 12,000 *g* for 20 min. The centrifuged solution could be used for mass spectrometry analysis (Zhu et al., 2022).

### LC-MS/MS analysis

The qualitative proteomics was analyzed by Easy-nLC 1,000 nl liquid chromatography (Thermo Fisher Scientific, Waltham, United States). The flow phase A was 0.1% FA-water, and the flow phase B was 0.1% FA-ACN, gradient elution: 0–25 min, 5%–9% B; 25–65 min, 9%–23% B; 65–75 min, 23%–32% B; 75–76 min, 32%–95% B; and 76–90 min, 95%–95% B. The flow rate is 400 nl min^-1^. Mass spectrometry was performed on a QE Plus mass spectrometer in the data-dependent acquisition mode, and specific details were according to our previous research (Ma et al., 2018).

### Protein identification and quantitation

The raw data obtained by mass spectrometry were imported into Proteome Discoverer software (version 2.5) for protein identification. The qualitative analysis of proteins was performed using MaxQuant software (version 1.5.6.5) with Uniprot Data (http://www.uniprot.org/, contains 20,359 proteins) (Cox and Mann, 2008). The parameters were set as follows: maximum of two missing protein sites; fixed modification: cysteine and iodide acetalization; variable modification: methionine oxidation and N-acetylation; mass deviation of fragment ions: 0.02 Da; and minimum detection value of peptide: seven amino acids. A maximum false positive rate of 1% was allowed. The analysis was developed according to the previous research (Wang et al., 2021).

### Bioinformatics analysis

The false-positive rate Q < 0.01, fold change>1.2, and *p*-value < 0.05 were regarded as differentially expressed proteins. After obtaining quantitative information, bioinformatics software analyzed the differential proteins to find the relevant biological pathways and network interaction information. The biological function of the protein was analyzed by using the gene ontology analysis database GeneOntology (http://geneontology.org/) and UniProt (HYPERLINK "https://www.uniprot.org/" \o "https://www.uniprot.org/"https://www.uniprot.org/) database, and the biological function enrichment analysis was carried out based on the DAVID (https://david.ncifcrf.gov), Kyoto Encyclopedia of Genes and Genomes (KEGG) (www.genome.jp/kegg/), and STRING (http://www.string-db.org/) databases.

### Enzyme-linked immunosorbent assay

The levels of relative proteins were detected by enzyme-linked immunosorbent assay (ELISA). The levels of IL6 and MCP-1 were determined by employing the ELISA kit (Thermo Fisher Scientific, Waltham, United States). The levels of TNF-α, ICAM-1, and VCAM-1 were measured using an ELISA kit (Nanjing Jiancheng Bioengineering Institute). All operations shall be carried out according to the manufacturer’s protocol.

### Statistical analysis

SPSS22.0 statistical software was used for statistical processing, and the measurement data were expressed by x ± s. One-way analysis of variance was used to analyze the data, and a *t*-test was used for group comparison. Means and standard deviations (SDs) were used to present the findings. *p* < 0.05 was considered to be statistically significant. The ROC curve was established by GraphPad Prism 8.

## Results

### Population characteristics

A total of 65 patients were recruited for this study, and 56 completed the 12-week treatment. A total of nine subjects fell off during the trial, with a fall-off rate of 13.8%. The baseline characteristics of the subjects are summarized in Table 1. In this study, the age and sex of the two groups were matched, and there were more male patients than female patients. There was no significant difference in clinical indicators and disease history at recruitment. More than 36.67% of the patients had hypertension, and a few had coronary heart disease and cerebrovascular disease history. The commonly used indicators in blood detection showed no significant difference between the two groups (*p* > 0.05).

**TABLE 1:** Baseline characteristics of the study participants.

	AR group (*n* = 35)	Con group (*n* = 30)	*p-*value
Year	67.03 ± 9.51	63.12 ± 10.20	0.080
Male (n%)	17 (56.67%)	20 (76.92%)	0.280
Clinical profile			
BMI (kg/m^2^)	24.66 ± 3.65	25.32 ± 3.54	0.765
Resting heart rate (bpm)	74.5 ± 7.78	75.5 ± 7.54	0.838
Systolic blood pressure (mmHg)	14 1.07 ± 14.68	139.35 ± 12.63	0.925
Diastolic pressure (mmHg)	82.00 ± 9.34	85.42 ± 9.30	0.158
Smoking history (n%)	14 (46.67%)	14 (53.85%)	0.499
Drinking history (n%)	12 (40%)	13 (50%)	0.523
Etiology, n (%)			
Hypertension (n%)	11 (36.67%)	11 (42.31%)	0.798
Diabetes (n%)	4 (13.33%)	9 (34.62%)	0.148
CHD (n%)	4 (13.33%)	3 (11.54%)	0.908
Cerebral vascular disease (n%)	5 (16.67%)	4 (15.38%)	0.992
Dementia (n%)	1 (3.33%)	1 (3.85%)	0.991
Laboratory			
WBC (×109/L)	6.26 ± 2.15	6.63 ± 1.38	0.609
LYM (%)	28.08 ± 7.15	29.26 ± 11.59	0.847
NEUT (%)	61.83 ± 8.27	60.76 ± 12.04	0.767
RBC (×10^12^/L)	4.51 ± 0.54	4.42 ± 0.42	0.597
HGB (g/L)	135.28 ± 14.48	136.80 ± 12.34	0.669
PLT (×10^9^/L)	220.69 ± 69.99	217.08 ± 47.98	0.779
ALT (U/L)	32.63 ± 27.53	36.07 ± 27.90	0.273
AST (U/L)	23.07 ± 9.57	24.85 ± 11.81	0.303
BUN (mmol/L)	5.77 ± 5.56	7.06 ± 10.28	0.380
Cr (μmol/L)	62.09 ± 18.19	65.76 ± 19.56	0.666
CK (mmol/L)	78.42 ± 53.64	88.70 ± 36.19	0.728
GLU (mmol/L)	7.14 ± 3.48	6.82 ± 2.25	0.399
HDL (mmol/L)	1.67 ± 2.29	1.13 ± 0.31	0.318
HCY (mmol/L)	16.39 ± 7.37	13.95 ± 4.88	0.139

### Outcomes of Astragali Radix

**FIGURE 2:**
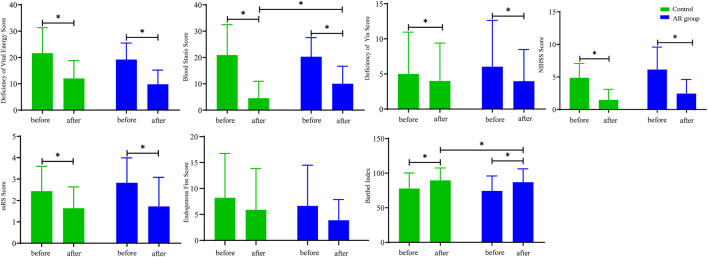
Indicators of symptom scores in the AR and control groups. AR: *n* = 30; control: *n* = 26. Before: represents the level of the patient before treatment; after: represents the level of the patient after 12 weeks of AR. Data are presented as mean ± SD. *p < 0.05.

After 12 weeks of treatment, the scores of deficiency of vital energy (DVE), blood stasis (BS), NIHSS, and mRS in the AR and control groups were significantly decreased (*p* < 0.05; Figure 2), and the endogenous fire score was decreased, whereas the BI score was increased (*p* < 0.05). Furthermore, the study found that compared with the Con, BS in the AR group was significantly decreased with a sharp increase in BI (*p* < 0.05). The scores indicated that AR could effectively alleviate the symptoms of patients with CI and protect the white matter to a certain extent, which may mainly improve the neurological function of the patients.

As shown in Table 2, there was no significant difference in TG, TC, and HDL-C between the two groups before and after treatment (*p* > 0.05); compared with the same group before treatment, AR treatment significantly reduced LDL-C as the control group (*p*<0.01).

**TABLE 2:** Comparison of the change in the serum lipid level between the AR and control groups.

	AR group (*n* = 30)	Con (*n* = 26)	*p*-value
TG (mmol/L)			
Before treatment	1.75 ± 1.06	1.74 ± 1.1	0.970
After treatment	1.67 ± 1.03	2.1 ± 1.13	0.261
TC (mmol/L)			
Before treatment	3.76 ± 0.82	3.42 ± 0.64	0.084
After treatment	3.58 ± 0.83	3.56 ± 0.57	0.935
HDL-C (mmol/L)			
Before treatment	1.12 ± 0.24	1.12 ± 0.27	0.966
After treatment	1.25 ± 0.26	1.15 ± 0.45	0.457
LDL-C (mmol/L)			
Before treatment	2.57 ± 0.7	2.1 ± 1.13	0.003
After treatment	1.97 ± 0.72**	2.04 ± 0.63	0.442

TC, total cholesterol; TG, triglyceride; LDL-C, low-density lipoprotein cholesterol; HDL-C, high-density lipoprotein cholesterol. *p < 0.05 and **p < 0.01.

### Safety assessment

Laboratory indexes were tested in this study, and there was no significant difference between the AR and the control groups (*p* > 0.05; see Supplementary Material S3). In addition, no significant adverse reactions were reported in both groups during the treatment period. See Supplementary Material S3 for details.

### Results of proteomics

#### Data quality control of quantitative proteomics

**FIGURE 3:**
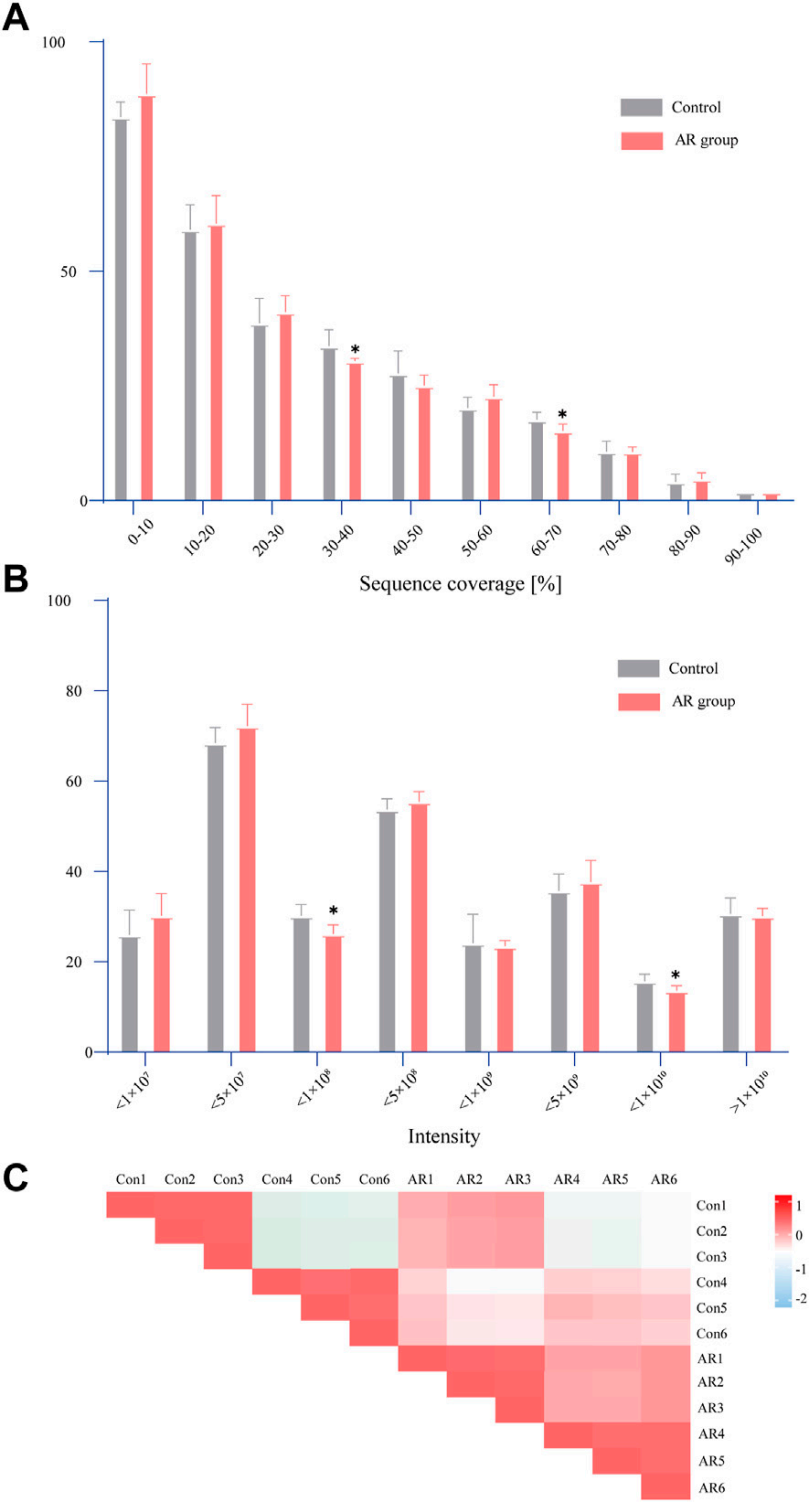
Statistical analysis of proteome data. **(A)** Distribution of sequence coverage of proteome data on different samples. **(B)** Signal intensity distribution of proteome data on different samples. **(C)** Pearson correlation analysis (the comparison between the two groups was conducted using an independent sample *t*-test, *n* = 6 per group, *p < 0.05).

As shown in Figure 3, the standard deviation of the same sample is very small, indicating that the data on the same sample have good repeatability. The analysis of the AR group and the Con discovered significant differences in the coverage of 30%–40% and 60%–70%. It can be seen from the signal intensity distribution diagram that there are significant differences between the AR group and the Con in different signal intensities of the two groups (such as <1×10^8^ and <1×10^10^). The Pearson correlation results illustrate a difference between the AR group and the Con. Interestingly, specific differences are also found among the six samples of each group, which the contrasts between males and females may cause. Details of the LC-MS data can be found in Supplementary Material S4.

### Identification of differentially expressed proteins

**FIGURE 4:**
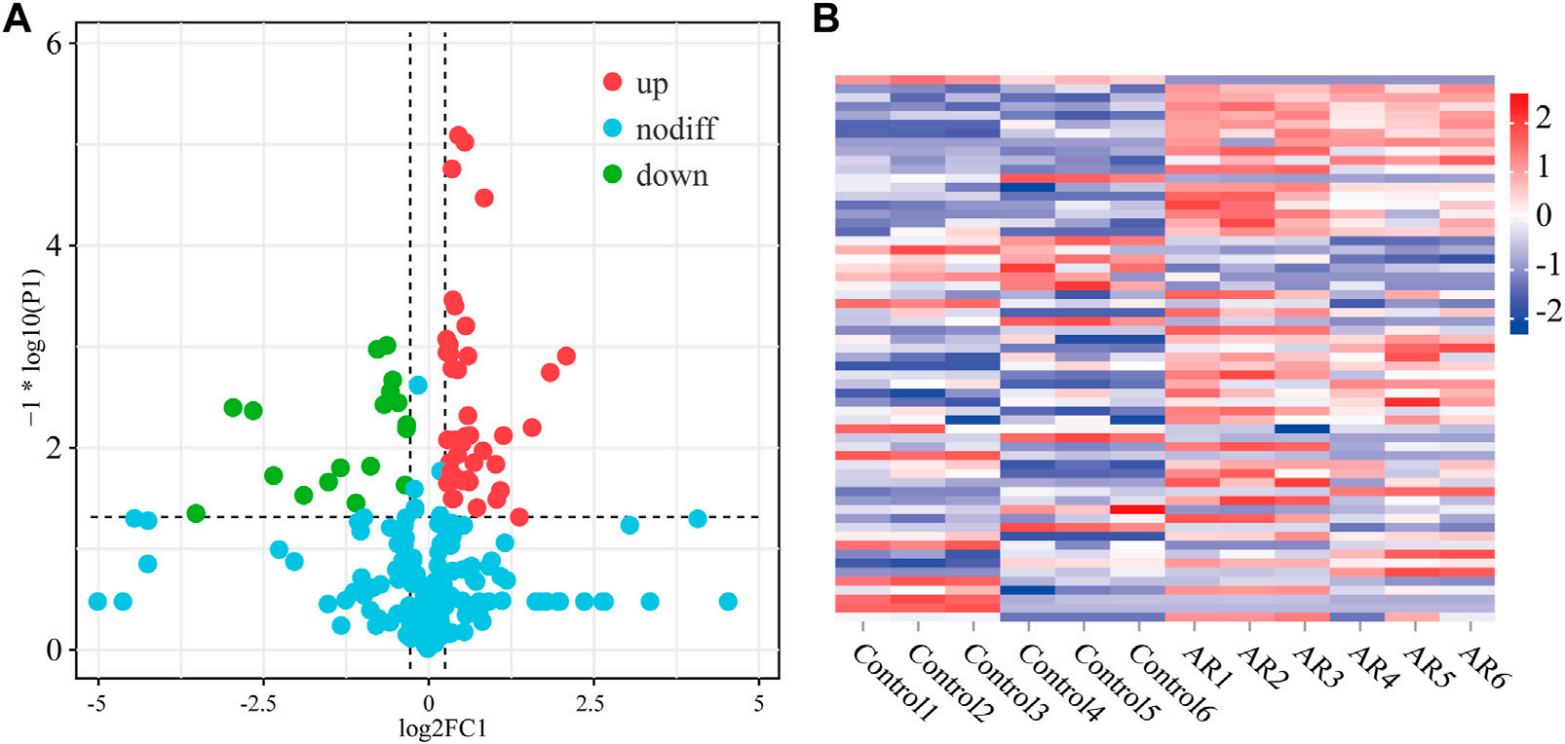
Differentially expressed proteins of the AR and control groups. **(A)** Volcano map of differentially expressed proteins of AR/Con. **(B)** Heatmap analysis of serum samples of the AR and control groups (*n* = 6 per group).

**FIGURE 5:**
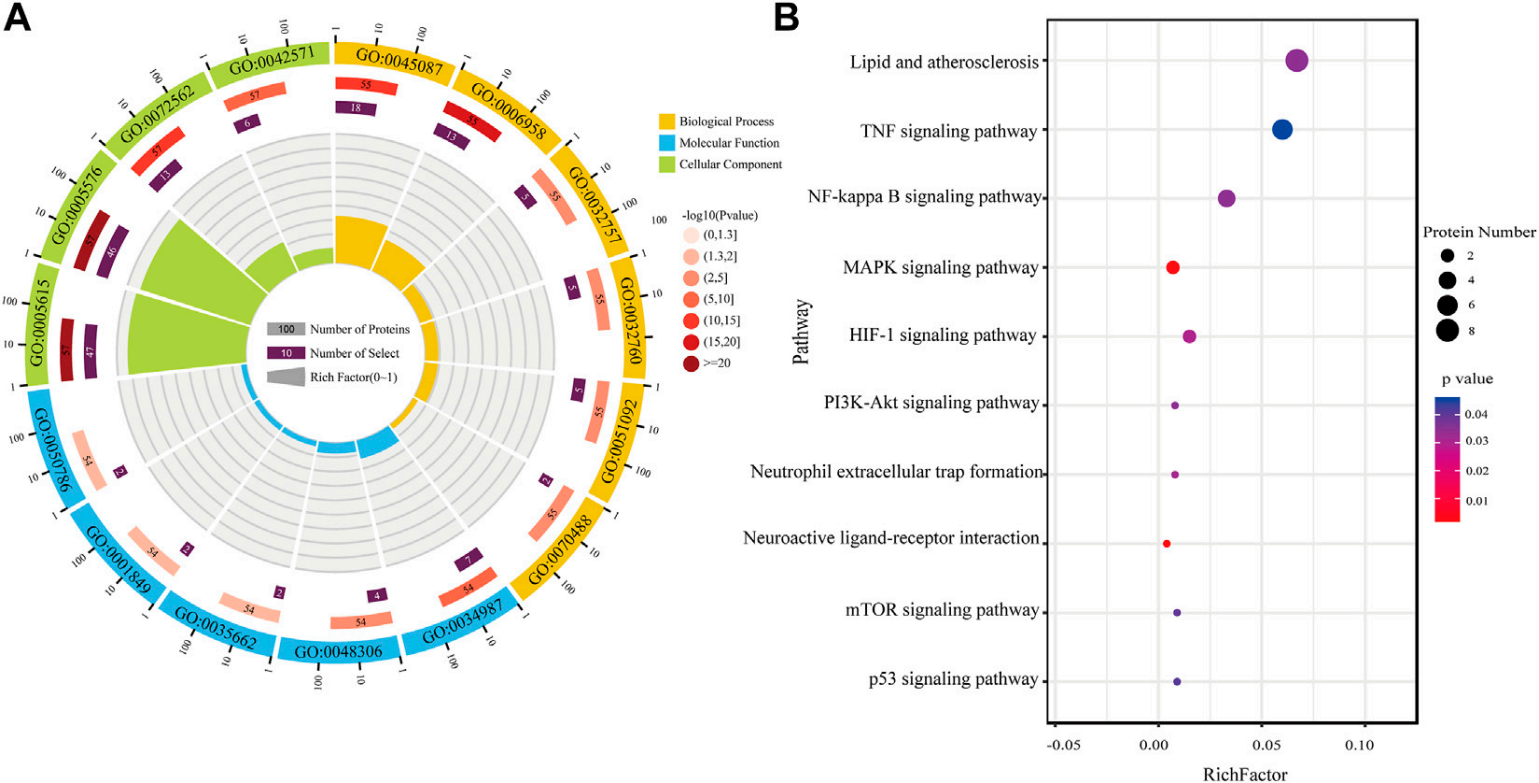
Functional enrichment analysis. **(A)** GO analysis of differential proteins in AR/Con. **(B)** KEGG analysis of differential proteins in AR/Con.

The difference in the protein expression level was more than 1.2 times a significant difference, and the differentially expressed proteins among the three groups were analyzed (Figure 4). In the volcanic map, green dots represent downregulated proteins, red dots represent upregulated proteins, and blue dots represent proteins with no significant difference. The horizontal axis is represented by log 2 (fold change), and the proteins with significant differences are distributed at both ends. The ordinate is represented by log10 (*p*-value). The larger the value, the clearer is the difference. Compared with the control, AR upregulated 43 proteins and downregulated 20 proteins. The heat map in Figure 5 shows the distribution of all the different proteins in the serum samples, and the colors in different degrees represent the protein content. It can be seen that the proteins show evident clustering in the two groups. The heat map results are similar to those of the volcano map. Compared with Con, more differential proteins in the AR group were upregulated.

### Functional classification of differentially expressed proteins

To understand the functional categories of differential proteins, the Gene Ontology and STRING databases were used for Gene Ontology (GO) analysis. The results were expressed using an enrichment circle map (Figure 5). The first circle from the outside to the inside is the enrichment classification. Different colors represent different classifications, including biological process (BP), molecular function (MF), and cellular component (CC). The scale with the number of proteins is outside the circle. The second circle is the number of background proteins and the *p*-value. The more the number of proteins, the longer the bar. The redder the color, the smaller the *p*-value. The third circle is the total number of proteins in each classification. The fourth circle represents the rich factor of each classification (Qie et al., 2020).

After the AR exerts efficacy in the organism of patients with CI, it may play a role in molecular function, including the immune system and energy metabolism-related functions such as immunoglobulin receptor binding (GO:0034987), Toll-like receptor four bindings (GO:0035662), complement component C1q binding (GO:0001849), and calcium-dependent protein binding (GO:0048306), through biological processes such as complement activation, classical pathway (GO:0006958), innate immune response (GO:0045087), positive regulation of tumor necrosis factor production (GO:0032760), and neutrophil aggregation (GO:0070488). All the differentially expressed proteins were at the extracellular space (GO:0005615), extracellular region (GO:0005576), and blood microparticle (GO:0072562).

### Cluster analysis of differentially expressed proteins

Based on the aforementioned ontology analysis, the biological functions of differential proteins were analyzed by KEGG. The rich factor indicated the ratio of differential proteins enriched to the number of all proteins in a pathway. The higher the rich factor, the higher the degree of enrichment. According to the value of the enrichment factor, the top 10 enrichment paths were selected for visualization. As the results indicate, the proteins regulated after treatment with AR in patients with CI were mainly related to biological processes such as the TNF-α signaling pathway, NF-κB signaling pathway, HIF-1 signaling pathway, neuroactive ligand–receptor interaction, and p53 signaling pathway. The study showed that AR might play a neuroprotective role in CI through anti-inflammatory and antioxidant properties, promoting neurogenesis and angiogenesis.

### Protein–protein interaction analysis of differentially expressed proteins

**FIGURE 6:**
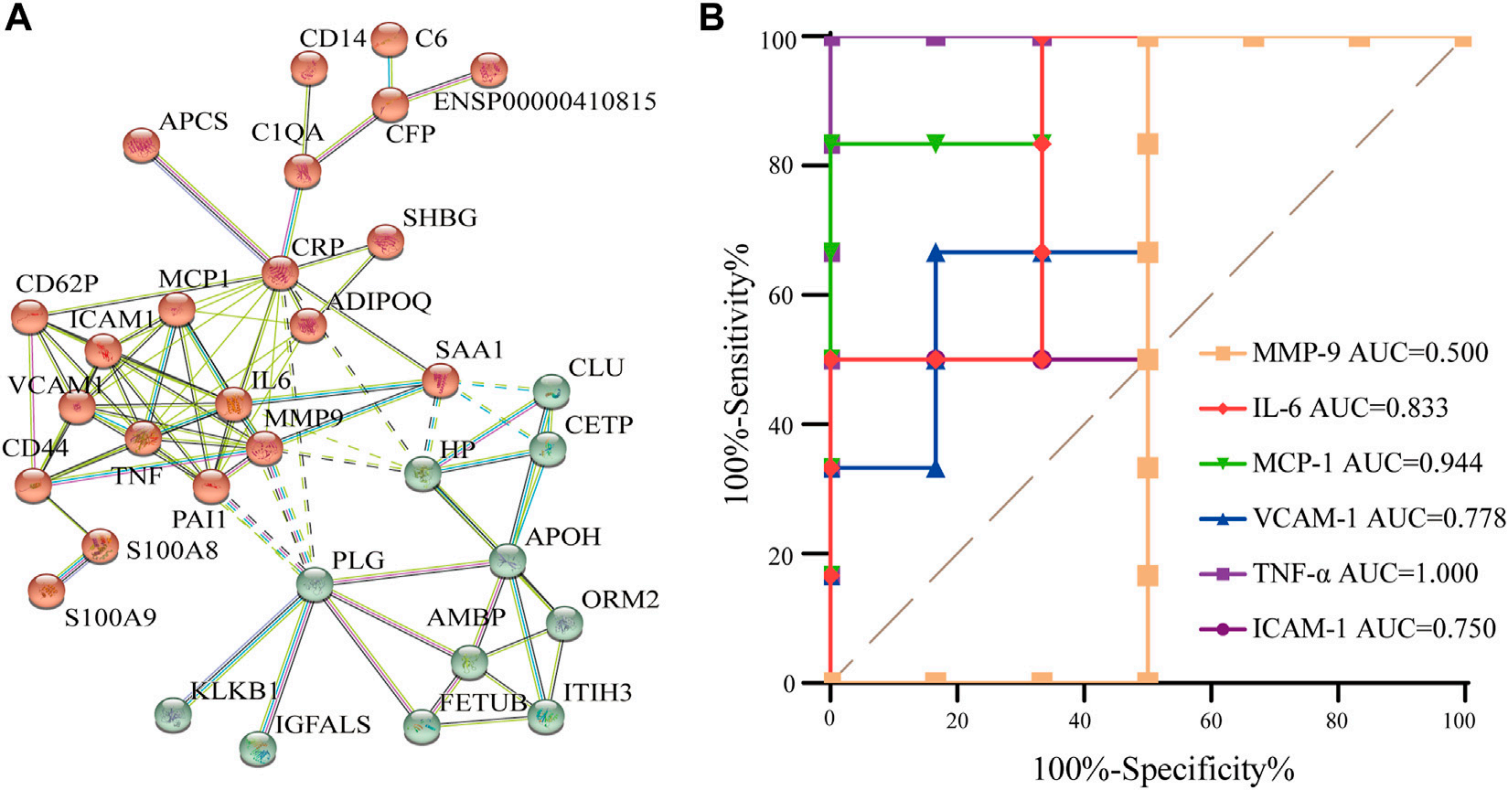
PPI analysis and the ROC curve of differentially expressed proteins. **(A)** PPI analysis of differential protein in AR/Con. **(B)** ROC curve of six potential proteins.

Proteins in the body usually interact with each other to complete a series of biological processes. Furthermore, the STRING database (http://string-db.org) was used to predict the interactions of enriched proteins in the pathway to identify key nodes in the development of AR therapy for CI. Aiming to improve the reliability of the PPI analysis, the confidence score was set at high confidence (≥0.700). Figure 6A demonstrates their interaction. In total, 32 proteins were detected and showed complex interactions with other proteins between the AR and the control. In Figure 6A, two protein clusters were demonstrated to be highly related to CI, and the red cluster contained more proteins; the interactions were complex and formed a network that represents the target proteins that AR may act on. Combined with the proteins in the top 10 KEGG pathways, IL-6, TNF-α, MMP-9, ICAM-1, VCAM-1, and MCP-1 are the central proteins of the PPI network, indicating that they have multiple interactions with other proteins and may be potential targets for AR.

### Origin prediction by the ROC curve

To verify the accuracy and specificity of the screening of potential target proteins, an ROC curve was established to judge the feasibility of prediction. As shown in Figure 6B, the area under curve (AUC) of IL-6, TNF- α, ICAM-1, VCAM-1, and MCP-1 is greater than 0.7, and the AUC of MMP-9 is less than 0.7, indicating that IL-6, TNF- α, ICAM-1, VCAM-1, and MCP-1 have good accuracy in the diagnosis of AR, and the five screened proteins have diagnostic abilities.

### Validation of the five candidate proteins

**FIGURE 7:**
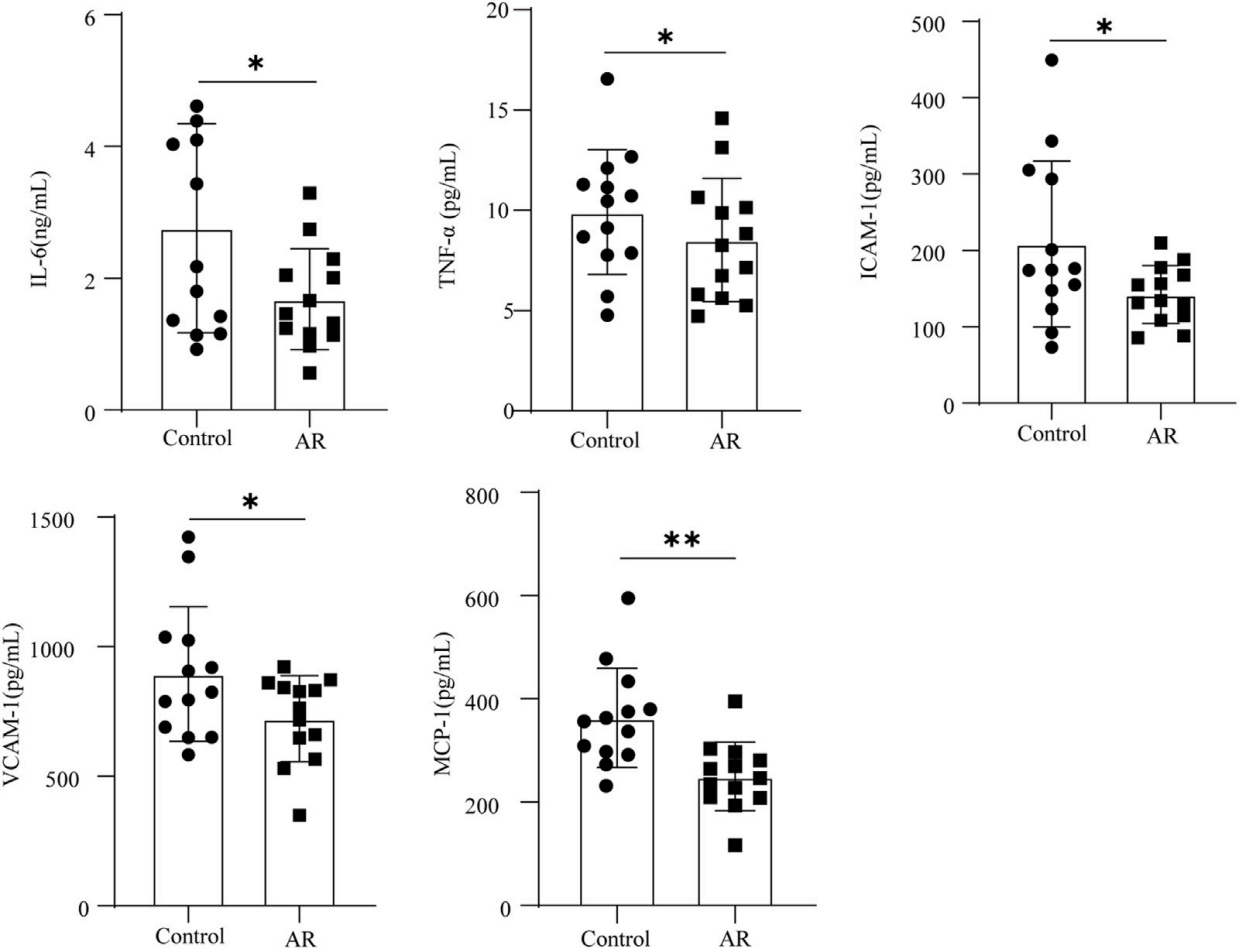
Protein content of the AR and control groups was determined by ELISA (*n* = 13 in each group). Data are presented as mean ± SD. *p < 0.05; **p < 0.01.

The aforementioned analysis results showed that IL-6, TNF-α, ICAM-1, VCAM-1, and MCP-1 may be potential candidate proteins for the AR treatment of CI. ELISA quantified the levels of these proteins in the serum, and the results are shown in Figure 7. Compared with the control, the levels of IL-6, TNF-α, VCAM-1, MCP-1, and ICAM-1 were significantly decreased in the serum of the AR group (*p* < 0.05, *p* < 0.01). IL-6, TNF-α, VCAM-1, MCP-1, and ICAM-1 might be potential target proteins for the AR to treat CI.

## Discussion

In this study, we assessed the effectiveness and safety of AR in treating CI for the first time through a randomized controlled trial. The combined results showed that the AR significantly improved the clinical symptoms and cardiac function of patients with CI, ameliorated energy metabolism, and regulated coagulation and neurological function. The results also showed that the scores of DVE, BS, NIHSS, mRS, and LDL-C significantly decreased with the treatment of the AR (*p* < 0.05), yet that of BI increased (*p* < 0.05). We inferred the therapeutic mechanism of AR may be related to the regulation of blood glucose and blood lipid levels, the improvement of neurological function and anticoagulant activity, and the reduction of the risk of thrombosis.

Furthermore, quantitative proteomics was used to further elucidate the mechanism of the AR. Comparison analysis revealed a significant quantitative alteration in protein with differential abundance between the two groups, resulting in 63 differentially expressed proteins in the AR group. Moreover, the bioinformatics analyses and validations revealed that the AR exerted the effects by acting on lipid and atherosclerosis, the TNF-α signaling pathway, NF-kappa B signaling pathway, and neuroactive ligand–receptor interaction. A further comprehensive discussion was conducted on the research results of the protein–protein interaction functional network, combined with the proteins in the top 10 KEGG pathways, and we discovered that IL-6, TNF-α, MMP-9, ICAM-1, VCAM-1, and MCP-1 were located in the center of the network with the treatment of the AR recipe, serving as a hub to interact with other proteins. The proteins mainly participated in biological processes of the immune system, including the TNF-α signaling pathway and NF-kappa B signaling pathway. The ROC curve and ELISA indicated that IL-6, TNF- α, ICAM-1, VCAM-1, and MCP-1 have good accuracy in the diagnosis of AR.

The formation of atherosclerotic lesions is a complex process, which often proceeds over decades. Several different factors contribute to the formation of atherosclerotic lesions. Atherosclerosis is the predecessor of stroke, which belongs to chronic vascular inflammatory reactions. Tumor necrosis factor *α* (TNF-α) is the main pro-inflammatory cytokine that contributes to the occurrence and maintenance of vascular inflammation. TNF-*α* induces the production of reactive oxygen species (ROS), thereby driving the redox reaction that constitutes “ROS signaling.” However, these ROS may also cause oxidative stress, leading to vascular dysfunction (Lamb et al., 2020). Atherosclerosis may be related to the increase in intracellular IL-6 levels. During the aging process, IL-6 signaling in bone marrow adipocytes increases, which may lead to the differentiation of hematopoietic stem cells toward bone marrow cells and increase the risk of gene mutations encoding transcription regulatory factors (such as TET2), which may lead to positive selection and expansion of hematopoietic cell clones. Clone results of bone marrow cells with TET2 mutation showed IL-6 and IL-1 β, which may lead to accelerated atherosclerosis (Tyrrell and Goldstein, 2021). Activation of the NF-kappa B signaling pathway may be associated with the aggravation of ischemic cardiocerebrovascular disease (Li et al., 2018). Previous studies have also shown that prescriptions of TCM including AR can inhibit the NF-kappa B signaling pathway and adjust blood lipid levels to treat atherosclerosis (Liu et al., 2020).

The TNF-α signaling and NF-kappa B signal pathways are closely related to inflammation and neuronal apoptosis, which are essential nuclear transcription factors involved in regulating cell differentiation and apoptosis in organisms and controlling inflammatory and immune responses. Inflammatory signaling is activated and completed by blood-derived leukocytes, which penetrate the brain during ischemia and produce inflammatory cytokines and proinflammatory mediators, including TNF-α, IL-6, and ICAM-1 (Xu et al., 2018). TNF-α is a common cytokine that promotes inflammation, induces the release of inflammatory cytokines, and promotes cell necrosis (Chen et al., 2019). Compared with the control, the level of TNF-α in the AR group significantly decreased after treatment (*p* < 0.05), which suggested that the neuroprotective effects of AR may be related to the inhibition of neurogenic inflammation caused by cerebral ischemia. As for ICAM-1, other studies defined the protein’s crucial role in the development of atherosclerosis by participating in the processes of inflammation and apoptosis (Patel et al., 2020). ICAM-1 is present in atherosclerotic lesions and is involved in their progress. In addition to its well-known role in white cell migration, ICAM-1 has now been proven to be able to transmit intracellular signals, leading to the rearrangement of actin to the skeleton and the activation of pro-inflammatory cascade reactions, thus making the inflammatory response continuous (Lawson and Wolf, 2009). In addition, AR can reduce the expression of ICAM-1 and VCAM-1, thereby reducing the activation of NF-kappa B in human endothelial cells. Over the years, MCP-1 has emerged as an important chemokine, playing a pivotal role in many CNS disorders (Joly et al., 2020; Singh et al., 2021). MCP-1 has been recommended as an early predictor of ischemic stroke (Bonifacic et al., 2016; Li et al., 2020). AR downregulates MCP-1 significantly, which may play a role by reducing the neurological impairments, inflammatory response, and ischemic infarct area. The aforementioned results suggest that AR may play a neuroprotective role in CI by reducing proinflammatory substances, thereby inhibiting the activation of the TNF-α signaling and NF-kappa B signaling pathways.

Our research still has some limitations and shortcomings. First, we only tested the serum proteins of the patient after treatment; so, we need to further examine the changes in serum proteomics and other time points throughout the entire process. In addition, more patients and more experiments may be needed to prove the aforementioned conclusion.

## Conclusion

In summary, this study found that AR can significantly improve the clinical symptoms of CI. In addition, based on the results of serum proteomics research of clinical patients with CI, we found that compared with control, AR upregulated 43 proteins and downregulated 20 proteins. Through analysis of protein functional enrichment, it was found that AR may act on IL-6, TNF-α, ICAM-1, VCAM-1, and MCP-1 and play anti-atherosclerosis and neuroprotective roles. This study explores the biological mechanism of the AR formula in treating CI at the protein level, providing a molecular basis for the clinical treatment of CI and related research of TCM.

## Data Availability

The mass spectrometry proteomics data have been deposited to the ProteomeXchange Consortium (http://proteomecentral.proteomexchange.org) via the iProX partner repository (Ma et al., 2018) with the dataset identifier PXD029388.

## Abbreviations

AR, Astragali Radix; AUC, area under curve; BP, biological process; BI, Barthel Index; BS, blood stasis; CC, cellular component; CI, cerebral infarction; DEPs, differentially expressed proteins; DTT, dithiothreitol; DY, deficiency of Yin; DVE, deficiency of vital energy; ELISA, enzyme-linked immunosorbent assay; FA, formic acid; GO, Gene Ontology; Con, control group; HDL-C, high-density lipoprotein cholesterol; ICAM-1, intercellular adhesion molecule 1; IAA, iodoacetamide; IL-6, interleukin-6; KEGG, Kyoto Encyclopedia of Genes and Genomes; LDL-C, low-density lipoprotein cholesterol; MMP-9, matrix metalloproteinase-9; MF, molecular function; MCP-1, monocyte chemoattractant protein-1; NIHSS, NIH Stroke Scale; PPI, protein–protein interaction; TG, triglyceride; TC, total cholesterol; TNF-α, tumor necrosis factor-alpha; VCAM-1, vascular cell adhesion protein 1.
