# Supplementary material for: Neuroprotective effect of Astragali Radix on cerebral infarction based on proteomics
Source: Front Pharmacol. 2023 Jun 9;14:1162134. doi: 10.3389/fphar.2023.1162134 (PMC10289882; doi:10.3389/fphar.2023.1162134)
Supplement: Supplementary file 3 [file DataSheet2.docx]

**Supplementary materials 2**

**Analysis of chemical metabolites of Astragali Radix (AR, Huangqi in Chinese)**

**1** **Materials**

**1.1 Instrument**

API 4000 QTRAP mass spectrometry system (ESI ion source, applied biosystem, USA); 1200 high performance liquid chromatograph (Agilent, USA); Positive Pressure-96 processor (waters, USA); KH-300E ultrasonic cleaner (Kunshan Hechuang Ultrasonic Instrument Co., Ltd.); MS1 Minishaker vortex mixer (IKA, Germany); Calibra precision pipette (socorex, Switzerland); AE240 precision analytical balance (metter, USA); MDF-0333 ultra low temperature refrigerator (Sanyo company, Japan)

**1.2 Standards and reagents**

Formononetin (3524, 98.3%), formononetin glucoside (3818, 98.1%), calycosin (6126, 98.6%), calycosin-7-O-β-D-glucoside(5240, 98.8%), astragaloside I (6107, 99.0%), astragaloside II (3824, 100.0%), astragaloside III (4053, 100.0%), astragaloside IV (6528, 100.0%), and cycloastragenol (4007, 98.2%) were purchased from Shanghai shidande Standard Technical Service Co., Ltd. Methanol (chromatographic purity, Fisher, LOT111773); purified water (Hangzhou Wahaha Co., Ltd., China).

**2 Experimental method**

**2.1 Preparation of standard solution**

Accurately weighed each reference substance, add 1.0 ml methanol, shaked and mixed evenly, and the preparation concentration was 1 μg/ml standard mother liquor. The formononetin, formononetin glucoside, calycosin, calycosin-7-O-β-D-glucoside, astragaloside I, astragaloside II, astragaloside III, astragaloside IV, and cycloastragenol were absorbed 100 ul respectively. And 100 ul methanol was added, shaken and mixed to form a mixture with a concentration of 100 ng / ml.

**2.2 Preparation of test solution**

The composition of Astragali Radix granules was 60 g of Astragalus membranaceus (Fisch.) Bge. var. mongholicus (Bge.) Hsiao, which were provided by China Resources Sanjiu Pharmaceutical Co., Ltd., and the batch number was 1812002C. Astragali Radix were ground into powder in a mortar, 4 g of powder was weighed and 20 ml of methanol was added, ultrasonic (40 Hz, 300 W) for 30 min, and then cooled with 0.22 μm microporous membrane filtration.

**2.3 Chromatographic and Mass spectrometry conditions**

**Chromatographic conditions:** injection volume 10 μL. The flushing mode of the injector was flush port, with a flushing time of 10 sec and a running time of 20 min; The column temperature box was 10 ℃, the flow rate was 0.2 ml/min, the mobile phase A was water (containing 0.05% formic acid), and the phase B was an equal proportion mixture of methanol and acetonitrile (containing 0.05% formic acid), elution gradient (0-0.2min, 20% B; 0.2-1.5min, 20%-85% B; 1.5-9min, 85% B; 9.01-20min, 20% B)

**Mass spectrometry conditions:** ESI source, the air curtain gas was 10 p.s.i., the gas GS1 in the source was 50 p.s.i, the gas GS2 in the source was 50 p.s.i, the ion injection voltage 5000 V, the temperature in the source was 600 ^o^C, the collision gas (CAD) medium, the detection method was positive ion detection, and the scanning method was response monitoring (MRM). The ion pairs used for quantitative analysis were shown in the Table 1.

**Table 1 MRM Parameters**

| Q1 MS (Da) | Q3 MS (Da) | Time (msec) | Name | DP (volts) | EP (volts) | CE (volts) | CXP (volts) |
| --- | --- | --- | --- | --- | --- | --- | --- |
| 447.1 | 285.1 | 50 | Calycosin-7-O-β-D-glucoside | 68 | 10 | 28 | 10 |
| 431.2 | 269.2 | 50 | Formononetin glucoside | 66 | 10 | 24 | 10 |
| 255.1 | 199.1 | 50 | Daidzein | 80 | 10 | 35 | 10 |
| 255.1 | 137.1 | 50 | Daidzein | 80 | 10 | 37 | 10 |
| 417.1 | 255.1 | 50 | Daidzein | 55 | 10 | 23 | 10 |
| 269 | 197.2 | 50 | Formononetin | 54 | 10 | 40 | 10 |
| 285.2 | 253.2 | 50 | Calycosin | 43 | 10 | 29 | 10 |
| 513.5 | 513.5 | 50 | Cycloastragenol | 200 | 10 | 5 | 10 |
| 891.7 | 891.7 | 50 | Astragaloside I | 220 | 10 | 5 | 10 |
| 869.4 | 143 | 50 | Astragaloside I | 100 | 10 | 30 | 10 |
| 849.5 | 669.5 | 50 | Astragaloside II | 230 | 10 | 66 | 10 |
| 807.5 | 807.5 | 50 | Astragaloside III | 220 | 10 | 5 | 10 |
| 807.5 | 495.5 | 50 | Astragaloside III | 220 | 10 | 75 | 10 |
| 785.5 | 143.2 | 50 | Astragaloside IV | 50 | 10 | 20 | 10 |
| 785.5 | 785.5 | 50 | Astragaloside IV | 50 | 10 | 5 | 10 |

**3 Results**

As shown in the metabolites characteristic diagram of Figure 2, eight metabolites were detected in the Astragali Radix, such as Formononetin , Formononetin glucoside, Calycosin, Calycosin-7-O-β-D-glucoside, Daidzein, Astragaloside II, Astragaloside III, Astragaloside IV, and Cycloastrage.


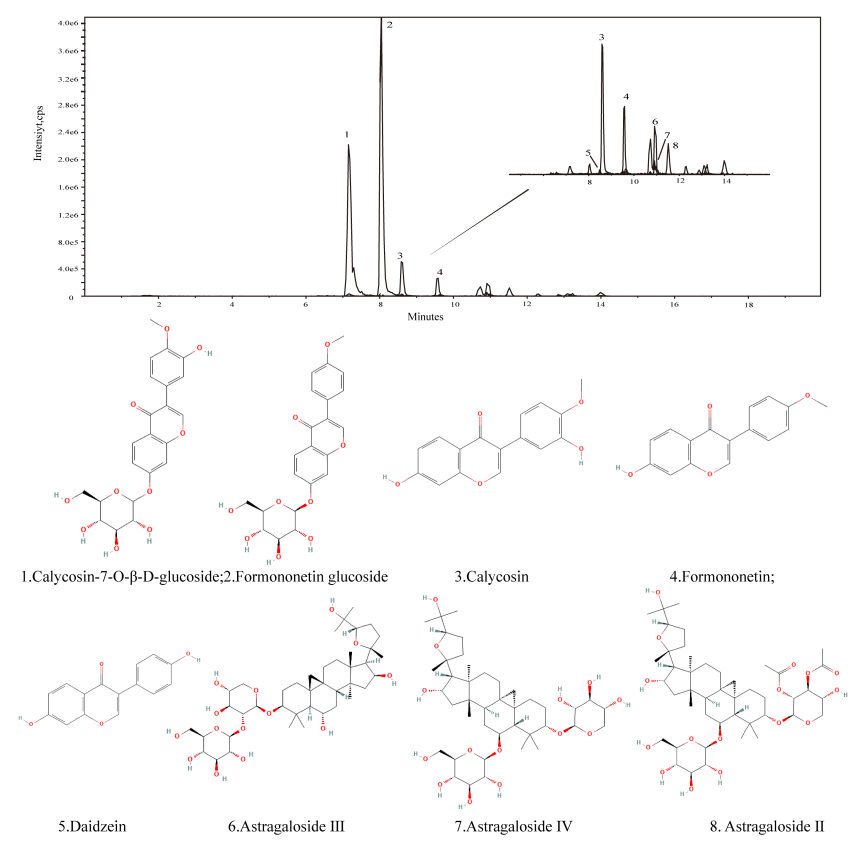


**Figure S2.** UPLC-Q-TOF-MS/MS Spectra and Structural Diagram of Chemical Metabolites of Astragali Radix
