# Supplementary material for: Neuroprotective effect of Astragali Radix on cerebral infarction based on proteomics
Source: Front Pharmacol. 2023 Jun 9;14:1162134. doi: 10.3389/fphar.2023.1162134 (PMC10289882; doi:10.3389/fphar.2023.1162134)
Supplement: Supplementary file 4 [file DataSheet1.DOCX]

**Supplementary materials 1**

**1 Diagnostic criteria**

- 1. **Western diagnostic criteria (refer to "Diagnostic Essentials of various Cerebrovascular Diseases" 1997) atherosclerotic thrombotic cerebral infarction:**

1. it often occurs in a quiet state;
2. there is no obvious headache and vomiting in most cases;
3. it develops slowly and progresses gradually or in stages;
4. there is a clear or mild disturbance of consciousness within 1-2 days after onset;
5. there are symptoms and signs of internal carotid artery system and / or vertebrobasilar artery system;
6. brain CT or MRI is responsible for infarction.
   1. **Diagnostic criteria of traditional Chinese Medicine (refer to the criteria for evaluating the Therapeutic effect of diagnosis of apoplexy drafted by the Encephalopathy Emergency Research Cooperation Group of the State Administration of traditional Chinese Medicine (for trial implementation) 1995)**

**Diagnostic criteria of apoplexy**

1. main symptoms: hemiplegia, dizzy consciousness, astringent or silent speech, abnormal hemianaesthesia, askew of tongue and tongue.
2. secondary symptoms: headache, vertigo, pupil changes, choking in drinking water, blindness, ataxia.
3. Acute onset, there are many inducements before the onset, and there are often premonitory symptoms.
4. the age of onset is more than 40 years old.

With more than two main symptoms, or one primary disease and two secondary symptoms, the diagnosis can be made by combining the onset, inducement, premonitory symptoms and age; without the above conditions, the diagnosis can also be made combined with the results of imaging examination.

**Stroke staging standard**

1. Acute stage: the onset is within 2 weeks, and the maximum length of the middle viscera is 1 month.
2. recovery period: 2 weeks to 6 months after onset;
3. sequela period: 6 months after onset.

**Syndrome differentiation standard of qi deficiency and blood stasis syndrome in traditional Chinese medicine**

1. main symptoms: hemiplegia, skewed tongue, unfavorable speech, abnormal partial sensation.
2. Secondary symptoms: pale complexion, shortness of breath, spontaneous sweating, dark complexion, or dark lips.
3. tongue image: the quality of the tongue is dull, the tongue has ecchymosis or collaterals at the bottom of the tongue, and the tongue coating is white and greasy or has tooth marks.
4. Pulse: the pulse is thick and thin.

The diagnosis of apoplexy is established, including two main symptoms and one secondary symptoms, which can be confirmed by combining tongue and pulse.

**2 Inclusion criteria**

1. in accordance with the western medical diagnosis of atherosclerotic thrombotic cerebral infarction and TCM syndrome of qi deficiency and blood stasis;
2. CT/MRI has a clear infarct;
3. the course of the disease is in the recovery stage (2 weeks to 6 months);
4. the NIHSS score is 3~22 points;
5. carotid ultrasound examination has evidence of atherosclerotic plaque;
6. between 35 and 80 years old;
7. the patient signed the informed consent form.

**3 Exclusion criteria**

1. transient ischemic attack (TIA);
2. cerebral embolism caused by rheumatic heart disease, coronary heart disease and other heart diseases complicated with atrial fibrillation; stroke caused by brain tumor, brain trauma, blood disease, etc.;
3. pregnant or preparing for pregnancy, lactating women;
4. complicated with serious diseases such as liver, bone, hematopoietic system, endocrine system, osteoarthrosis and mental illness;
5. the following laboratory indexes are abnormal: (1) abnormal renal function (serum creatinine is greater than the normal value); (2) more than 50% of the upper limit of glutamic pyruvic transaminase; (3) arrhythmias of clinical significance;
6. those who are known to be allergic to the ingredients of this drug and allergic constitution;
7. those who have participated in clinical trials of other drugs;
8. those with active ulcers and bleeding tendencies.
